# Waste Pickers and Their Practices of Insurgency and Environmental Stewardship

**DOI:** 10.1177/10704965211055328

**Published:** 2021-10-20

**Authors:** Jutta Gutberlet, Santiago Sorroche, Angela Martins Baeder, Patrik Zapata, María José Zapata Campos

**Affiliations:** 1 Department of Geography, 8205 University of Victoria (UVic), Victoria, BC, Canada; 2 Consejo Nacional de Investigaciones Científicas y Técnicas (CONICET), Centro de Innovación de los Trabajadores, 62873 Universidad Metropolitana para la Educación y el Trabajo, Sarmiento, Buenos Aires, Argentina; 3 Emeritus Faculty at the Department of Biology, 369805 University Foundation Santo André (FSA), Santo André, Sao Paulo, Brazil; 4 School of Public Administration, 3570 University of Gothenburg, Gothenburg, Sweden; 5 School of Business, Economics and Law, 83210 University of Gothenburg, Gothenburg, Sweden

**Keywords:** sustainability, waste, waste pickers, insurgency, environmental stewardship

## Abstract

Informed by different grassroots learning and educational practices engaged in waste management, and drawing from the concepts of insurgent citizenship and environmental stewardship, we examine the role of waste picker organizations and movements in creating new pathways towards more sustainable environmental waste governance. Two case studies (Argentina and Brazil) demonstrate how waste pickers inform and educate the general public and raise the awareness of socio-environmental questions related to waste management. Different educational practices are used as strategies to confront citizens with their waste: to see waste as a consumption problem, resource, and income source. Our paper draws on grassroots learning (social movement learning and insurgent learning) and education (stewardship) aimed at the transformation of waste practices. We argue that waste pickers play an important role in knowledge production promoting recycling, in landfilling less and recovering more resources. We conclude that waste pickers act as insurgent citizens and also are environmental stewards.

## Introduction

Solid waste is a significant urban problem, receiving increasing attention by the public and government. In many cities in the global South, municipal waste is still primarily landfilled or dumped. Often, peripheral neighborhoods do not have regular garbage collection and the locals dump or burn their waste, if it is not collected by informal or small-scale waste entrepreneurs and waste pickers (Gutberlet et al., 2016, 2017). Most cities in Brazil and Argentina do not yet have a widely installed official recycling program in place. Only recently, some municipalities in Brazil have established contracts with waste picker cooperatives, responsible for selective waste collection which sometimes provides them with opportunities to engage in educating households about recycling. In Argentina, waste picker cooperatives engage in environmental education activities to improve formal selective waste collection, yet with very little recognition by the local government. Gille (2013) argues that all these developments reveal an emerging resource recovery waste regime, linked to policy shifts that promote resource recovery over disposal in landfills. No significant policy changes and implementations, however, have been materialized in terms of avoiding waste generation or even reducing the generation of materials that are not recyclable and will become waste once discarded. Ideas of consumerism, capitalism, economic growth, and global market primacy continue defining the socio-material relations of production, consumption, and waste (Corvellec, 2016).

Grassroots initiatives in the global South are challenging the current waste regime by rising socio-environmental concerns in waste management (Zapata & Zapata Campos, 2018). Recycling is not the solution to the waste problem, but it is rather a myth or a societal trap, that to some extent further reiterates the status quo of wasteful consumption and indiscriminate discard with no decreasing effect on the total waste production (Hird et al., 2014; Lepawsky, 2018; MacBride, 2012). Beyond regulations, education can play a significant role in shifting habits, lifestyles and in promoting policies towards more sustainable behaviors, processes, and practices (Asmawati et al., 2012; Boyes & Stanisstreet, 2012). In this paper, we argue that waste pickers can fulfill the task of building environmental awareness among the public for recycling. The educational processes we discuss build on the social learning of waste pickers, who have engaged with other waste pickers in promoting popular learning on waste prevention and resource recovery in the wider community. Wenger introduces communities of practice as a social learning system. “Arising out of learning, it exhibits many characteristics of systems more generally: emergent structure, complex relationships, self-organization, dynamic boundaries, ongoing negotiation of identity and cultural meaning, to mention a few” (Wenger, 2010, p. 179–180). Communities of practice are part of larger social systems involving many other actors and communities, institutions, movements, projects, or different forms of associations. However, the social learning within these communities is equally shaped by their specific situated institutional, political, or cultural contexts, which makes them also vulnerable to intrinsic power plays. Wenger further reiterates, “[t]he concept of community of practice yields an inherently ‘political’ view of learning, where power and learning are always intertwined and indeed inseparable” (2010, p. 190). Social learning theory recognizes the agency in learning and sees practice as active production of identity, a response to power, not as an outcome of it (Foley, 1999).

Waste pickers organize in cooperatives, associations, or unions to participate in waste management, providing different services, for which they want to be recognized and remunerated. Organized waste pickers are part of a community of practice. They meet regularly in cooperative assemblies, network meetings or other meetings, workshops, or conferences, where they can share their work experiences, expand their capacities and use their agency for advocacy. They promote recycling in their communities, sometimes by engaging in educational tasks, such as informal conversations with households, presentations at schools or community centers, or through participating in formal programs linked to environmental education. Some scholars argue that these grassroot initiatives and networks have turned into the new social movements of the urban poor that, intentionally or not, challenge the nature of the state, local governments, and civil society (Gutberlet, 2010; Mitlin, 2008; Zapata Campos & Zapata, 2012). We agree that waste pickers reveal what James Holston (2009) calls “insurgent citizenship,” a phenomenon of new urbanism emerging specially among subaltern populations in the peripheries of the cities in the global South, where individuals organize in movements and use the urban space as strategic terrain for making claims on the state, to address social and economic inequalities. This is particularly apparent in the movement against waste incineration, spearheaded by the worldwide alliance of grassroots groups, non-governmental organizations, and individuals in over 90 countries, called Gaia (Gaia, 2021). In Brazil, the national movement of waste pickers (MNCR, Movemento Nacional de Catadores de Materiais Reciclaveis) is one of the key organizers in the anti-incineration movement, together with regional and local networks of waste picker cooperatives and NGOs.

Waste pickers benefit from what Hall et al. (2005) call “‘social movement learning’, or learning by people who are part of a social movement and learning by individuals outside of a social movement as a result of the actions taken or simply by the existence of social movements” (Hall et al., 2005, pp. 584–589). Learning happens informally, and is often improvised and uses popular knowledge as much as acquired technical knowledge. Eyerman and Jamieson (1991) recognize the creative and central role of learning processes in what they call “cognitive praxis.” Waste pickers educate out of their everyday cognitive working praxis. Interacting with waste generators and manipulating recyclable waste on a daily basis exposes them to detailed practical learning on waste related issues. These grassroots knowledges are shared within their communities of practice during opportunities such as meetings, workshops, or other interactions as they surface.

This paper connects the literature on social movement learning with the work on environmental stewardship, to be introduced in the next section of this paper, by examining the role of organized waste pickers, part of a social movement, in creating new pathways towards more sustainable environmental governance and a more just society.

The paper is informed by the educational practices of waste picker organizations in Brazil and in Argentina, which the authors have accompanied for more than 10 years. These initiatives constitute important innovation spaces for sustainability and social justice (Brown & Vergragt, 2008). Addressing waste related challenges (e.g., the lack of regular recycling programs in the city and waste incineration) and bringing diverse knowledges into circulation (particularly from waste pickers or the public) creates the potential for innovations that stimulate and support interventions that improve waste management (e.g., door-to-door household waste collection and education for wasting less). The possibility of engaging mainstream policy-makers with the grassroots still remains a difficult task (Smith et al., 2014). However, in the cases presented here, we will argue, a bridging dialogue can be established between waste pickers and other stakeholders (e.g., government, the public, and universities). These examples introduce certain forms of innovations, tackling personal waste behaviors, the waste management system, and collective policy-oriented transformations to increase the rates and quality of recycling.

In the next section, we introduce the theoretical framework bringing together ideas on social movement learning (Hall et al., 2005) and environmental stewardship (Bennett et al., 2018) to contribute to the discussion on socio-economic and environmental aspects of waste. We will use structure and agency to analyze waste management education, discussing perspectives on relations, rationales, and resources. We then provide reflections on our methodology and present the two case studies on grassroots interventions in environmental education and insurgency. Concluding remarks will touch on the role of these educational actors and insurgent citizens who through their narratives address broad ideas on urban sustainability.

## Theoretical Insights

Discourses on waste vary significantly, with narratives drawing from landfilling as the solution in the past, to presently landfilling less and recovering more (Corvellec & Hultman, 2012). Increased recycling but also expanding incineration are part of the “landfilling less” narrative and are also connected to the understanding of “waste as a resource.” In the global North, waste incineration (waste to energy) has been seen as alternative to landfilling, despite the complex environmental impacts it generates (Corvellec et al., 2013; Demaria& Schindler, 2016), particularly in view of climate change. Without denying the economic value of waste, nor the need to reduce landfilling due to its environmental hazards, the new “wasting less” narrative becomes a multi-entry narrative that connects the social critique of an unsustainable consumption with the economic rationality of using resources effectively and making profits through their recovery (Corvellec & Hultman, 2012). Although it may sound contradictory, this political practice allowed waste picker organizations to dispute both the recognition of their work and, also, the consideration of it as an environmental service. On the other hand, when their work can be carried out correctly, the selective waste collection and separation for recycling directly results in less waste since these recovered materials are reinserted into production processes and are not buried or incinerated.

People have always found a way to extract value from specific waste (O’Brien, 2008; Strasser, 1999; Sjöstrand, 2014). Particularly in cities in the global South, waste pickers have been reclaiming those materials since long and are still doing so (Fahmi & Sutton, 2010). Official waste management in the global South considers primarily the collection and disposal of municipal waste at landfills or dumps. There are very little formal recycling programs for household waste in place and most materials are recovered by the informal sector (Kaza et al., 2018).

Organizing collectively, for example, into cooperatives, associations, networks, or social movements provides opportunities for empowerment and use of agency. In the case of waste pickers, their level of organization has allowed them to expand their knowledge and has already created significant changes and successes for the waste pickers. We discuss learning as “social movement learning,” describing how the organization of waste pickers contributes to social learning, which then frames educational processes to improve recycling.

Waste management education has been portrayed for the global North as a governmental tool used to discipline and normalize citizens into the dominant waste narrative of the prevailing waste regime, which currently worldwide depends on consumerism and throw away practices (Zapata & Zapata Campos, 2018). From that perspective, educating citizens to separate and to recycle ensures that waste management continues to be framed largely in terms of technological innovation, jurisdiction, and diversion practices, as is the case in many global North countries (Hird, 2013). Educating towards specific waste management forms can also create notions of citizenship, progress and even strengthen the understandings of statehood, and nationalism. For example, public educational discourses support the idea of being a good citizen, by recycling and having thus solved the waste problem (Hird, et al., 2014).

In the global South, the absence of waste collection and poor waste management practices as well as the lack of education often cause severe environmental and public health problems. Informal neighborhoods, which often expand over large areas, are not serviced by formal waste collection (Gutberlet et al., 2017). When looking for alternatives, too often the official narrative supports technological solutions from the global North, such as Waste to Energy to address the increasing quantities of waste being generated in the cities. In Argentina and Brazil, this process can be traced back to at least 45 years, when the construction of proposed sanitary landfills was imposed, mainly by multilateral organizations, as the hegemonic model of waste management in the region, (Sorroche, 2016a) and which, in recent years, has resurfaced in different proposals to establish incineration systems. In the transfer of these “Northern” technologies and waste management strategies, the “Southern” everyday context situated in local culture, environment, and politics is disregarded (Gulrajani & Swiss, 2019; Gutberlet et al., 2020; Troschinetz & Mihelcic, 2009).

In Brazil and Argentina, part of the waste pickers organize in cooperatives, associations, unions, federations, and networks and form social movements which provides new formulations on how to deal with waste and how to promote participatory waste management, disrupting existing assumptions about them that have sustained stigma and marginalization. The struggles of waste pickers, usually the most impoverished and socially excluded citizens, have also generated new movements of insurgent citizenship based on their claims to have a right to resources and spaces in the city as commons (Dias, 2016; Zapata & Zapata Campos, 2015). Holston has studied the citizen power and social justice struggles with the “entanglements of these insurgent urban citizenships” in different countries, including Brazil, particularly related to the homeless movement and the strive for housing in a political system of inequality and violence (Holston, 2008, 2009). In Argentina, high level of unemployment was the driver for popular struggles towards takeover and worker-managed factories in the late 1990s or, more recently, the organization of waste pickers into a social movement (Parizeau, 2015; Sorroche, 2016b).

The idea of resistance and manifestation is central to the organized waste pickers whom we identify as “insurgent citizens.” Waste pickers and their allies create new spaces of active citizenship, according to Miraftab (2004) “invented spaces,” to discuss and strategize interventions in waste management. In 2000, for example, waste pickers in São Paulo created the *São Paulo Recycling Forum*, and, in 2001, waste pickers formalized the organization of the National Recyclers’ Movement (MNCR) (Dias, 2011). The most important new spaces created by the waste pickers, since 2008, are networks (*Redes*) and inter-networks (*Inter-Redes*). In the case of Argentina, in 2011, the Argentine Federation of Waste Pickers (*Federación Argentina de Cartoneros, Carreros y Recicladores*) was created, which is now part of the Confederation of Workers of the Popular Economy (*Confederación de Trabajadores de la Economía Popular*). The Confederation brings together waste pickers, street vendors, small farmers, workers in recovered factories, and textile workers.

Through these organizations waste pickers have become forces and a movement acting outside the state. Friendly and Stiphany (2019, p. 275) describe insurgent movements as “those that manage to innovate amid the necessity of coping with urban inequalities.” The driving forces of insurgencies have to be located in the realm of civil society and in their daily life, originated from forces acting against the dominant political system. In opposition to the results of neoliberalism and particularly emerging out of urban peripheries, subaltern populations confront the regimes that have created the huge disparities and inequalities between the impoverished peripheries and the urban elite centers that benefit from their services and their poverty (Holston, 2009). Miraftab (2009) describes these citizen practices as insurgent, counter-hegemonic, transgressive, and imaginative. Waste picker organizations have become deeply involved in practices of collective action, empowering their members to challenge established assumptions about them being uninformed, mostly ignorant and incapable of knowledgeable decision-making (Holston, 2008). Leaders who used to work in the streets or dumps have used their agency to reconfigure power relations enabling them to partake in policy decision-making in waste management. The social movement learning has allowed them to “step out of the futures expected for them, and make a life that is totally unanticipated, a life for which there is no obvious preparation or eligibility" (Simone, 2010, p. 137).

Learning is a cognitive process that can take place in informal or incidental ways, in specific social contexts and can occur purely through observation or direct instruction. The learning can be enhanced when controversies are viewed and engaged in. The different world views of the collaborating actors then become assets in the process of transformation. Often starting out with conflicting world views and experiences, over time the different parties reach mutually agreeable problem definitions and develop solutions to questions and new products, or they develop practices based on reordered priorities and alternative values. These knowledge sharing interventions among members of the social movement (in meetings, conferences, or actions) or with other interlocutors (government agents, businesses, NGOs, academia, etc.) can be intensive and empowering for the participants.

The spaces where waste pickers act had to be conquered over many years and with persistent struggle. In some cases, waste picker organizations have a close dialogue with local politicians, advancing the movements’ agenda of inclusive waste management. Yet, as Holston notes, “the result is an entanglement of democracy with its counters, in which new kinds of urban citizens arise to expand democratic citizenships and new forms of urban violence and inequality erode them” (Holston, 2009, p. 246). Particularly, governmental change and political discontinuity, often come with a dismantling of the previous politics that had supported the waste pickers’ work (Zapata Campos & Zapata, 2014). That is one of the reasons why progress is being slow and development seems to sometimes go backwards.

Often waste pickers actively engage in environmental stewardship practices, through which they seek to transform the politics of waste management, granting them fair remuneration for their services. Different circumstances define whether and how individuals or groups become involved in stewardship activities. Bennett et al. (2018) differentiate between two broad analytical categories: “intrinsic motivations” (ethics) and “extrinsic motivations” (incentives) for engaging in environmental stewardship. These motivations can also be understood as “rationales.” Social relations, bodies, or informal networks can provide reinforcement and the rationales for local collective actions, generate resources or facilitate learning for stewardship, as oftentimes discussed in natural resource management situations, for example, small-scale artisanal fisheries (McConney, et al., 2014; Medeiros, et al., 2014). Whether the capacity for individuals and groups to engage in stewardship activities becomes empowered or constrained depends on different factors and assets that provide the capacity to enable these relations. Much of the success of stewardship initiatives depend on the level of organization as well as the local government support to involve grassroots actors, for example, in the selective waste collection. The continuity of door-to-door collection—which is an opportunity to practice environmental stewardship, by educating the households—most often depends on the continuous support by the government, for example, the city providing a truck for the collection, or remuneration for the amount of materials collected and separated. Stewardship, then, becomes a central tool for the political struggle for recognition of the work of waste pickers, realizing that it is not only their livelihood that ensures the activity but also the care of the environment in contexts where there are no public policies aimed at waste recovery. This dialogue with consumers makes it possible to generate awareness about separation at the source and, in this way, improve the daily working conditions of the waste pickers, which results in less waste sent to landfills and greater amounts of materials being re-integrated into the production processes of the circular economy.

Bennett et al. (2018, p. 601) describe how “procedural considerations, such as inclusion of stakeholders, participation in planning, social learning, knowledge co-production, cooperative management, trust building, negotiation, and conflict resolution, can also enable the effective stewardship of resources” (see also Jupiter et al., 2014; Lockwood et al., 2010; McConney et al., 2014; Turner et al., 2014). We argue that, particularly in the absence of any state program on environmental education, grassroots organizations can fill that gap in the community of waste management education, resulting in the promotion of door-to-door selective household and business waste collection or resulting in the creation of new small-scale waste organizations turning into institutional entrepreneurs in waste collection and diversion (Gutberlet, 2016; Gutberlet et al., 2017). Organized waste pickers have a distinct understanding of waste and waste management, situated in their everyday learning about waste. They form their own narratives and public discourses on waste.

In these cases, the individuals or groups make use of their agency and the existing structure to act as socio-environmental stewards. We take the definition provided by Bennett, et al. for local environmental stewardship, as “…the actions taken by individuals, groups or networks of actors, with various motivations and levels of capacity, to protect, care for or responsibly use the environment in pursuit of environmental and/or social outcomes in diverse social–ecological contexts” (2018, p. 597). The authors define stewardship assets or categories of assets that provide capacity to enable local environmental stewardship. The capacity and rationales depend on available resources, socio-economic status, race, gender, level of access to education, political will, and other aspects that allow for social inclusion. Local community assets and broader governance factors, such as public policies and funding are also involved.

## Research Methodology and Epistemology

This article builds on research which was conducted by the authors in São Paulo and in Buenos Aires. Three of the authors have a long standing relationship with the waste pickers in the region and can draw on many years of participatory research with waste pickers. In both cases, the authors work with participatory and action oriented research frameworks, embedded in an epistemological understanding of knowledge being co-generated with the participants during the research process. The authors were only instrumental in helping organize and facilitate the meeting space, and systematize the observations, voice recordings, and additional information collected during the research. In both cases, waste pickers were involved from the beginning in the design and implementation of the interventions. It is the authors’ understanding that participatory research is praxis oriented and transformative. Early on in the research process, during meetings with waste pickers and informal conversations during visits to the cooperatives, waste pickers identified the need to work on interventions or actions that would support the de-stigmatization of waste pickers, and improve the work in selective waste collection programs, advancing the level of knowledge amongst waste pickers and in general in the community.

This grassroots–academic collaboration (Gaudry, 2015) is based on critical social theory and radical pedagogies that are committed to provoking social and political change (Brandão, 1987; Freire, 1997; Santoro Franco, 2005; Thiollent, 2008), through meaningful dialogue. We take feminist and decolonizing lenses, valuing cultural and community identity as a building block for empowerment (Frisby et al., 2009). Doing research with and by people, rather than research on people has the potential to raise consciousness, awareness, and self-esteem of all participants, and it further helps identify needs, which can be addressed through the research process (Kindon, 2016).

Participatory action research (PAR) promotes mutual involvement, personal growth, and empowerment of the participants through the research process (Amauchi et al., 2021). Researcher and participants are actively involved in developing the goals and methods for collection and data analysis, as well as implementation of the results that will promote social change (Kidd & Kral, 2005). Particularly in the case of Buenos Aires, the original intervention has now become a long-term strategy which is still occurring. In the case of São Paulo, the intervention has been replicated several times and has been picked up by individual waste pickers who have incorporated the experiences from the intervention into their everyday praxis.

Participatory and empowering approaches are becoming more widespread in environmental and social studies, involving citizens as scientists, co-generating knowledge (Pandya, 2012). Participatory action research is dialogical and proactive (Thiollent, 2008). It seeks a reciprocal appreciation of co-researchers’ knowledge and skills (knowledge co-production), “going beyond the traditional scientific orthodoxy of positivist research… with the view that people need the opportunity to learn through their own experiences rather than be ‘objects’” (Green, 2008, p. 82–83).

An emphasis on emancipatory transformation is often implicit in the tools applied in PAR, and interventions can transform the lives of the participants. We have used workshops, general assemblies, participant observation, and citizen scientists approaches to work through the interventions. Throughout the application of these tools, we documented, transcribed, and systematized the information for data analysis, making sure to incorporate the feedback received from the participants. The specific approach and methods applied will be introduced for each of the two case studies in the following sections.

## Case Studies: Everyday Experiences in Education on the Reduction, Reuse, and Recycling

Next, we will introduce the empirical studies, conducted in Argentina and Brazil, focusing on the similarities in terms of structure and agency of the main protagonists involved in the activity of insurgent citizens, educating for environmental stewardship.

### Building and Expanding Environmental Education Programs for Female Waste Pickers in Buenos Aires, Argentina

This case draws on ethnographical research, based on PAR, that one of the researchers conducted with the National Waste Picker Federation (Federación Argentina de Cartoneros, Carreros y Recicladores—FACCyR) since August 2016. The key objective of this research was to develop systematized data that shows the developments of the federation that let the cooperatives to become part of the Integrated Solid Waste Management program. In Argentina, waste management is organized by the local governments, and waste picker cooperatives in Argentinian municipalities often struggle to be part of the local waste management system, managing the recyclables materials.

*Anuillan* (which means “decisive women” in Mapuche, one of the local indigenous languages) is a recycling cooperative composed mostly of women. The cooperative is also a member of the excluded workers movement (*Movimiento de TrabajadoresExcluidos—MTE*) and of the FACCyR, which is affiliated to the Workers Confederation of the Popular Economy (*Confederación de Trabajadores de la Economía Popular—CTEP*), in Argentina.

In 2014, *Anuillan* and the Recycling Board of the city of Buenos Aires (DGREC) decided to start an environmental stewardship program. Even though, waste separation is mandatory in the city of Buenos Aires since 2002, there was no state program in place to introduce or to reinforce the recycling program as a form of household waste management. The government did not encourage resource separation, while the waste pickers continued to access open curbside garbage bags containing mixed waste, in order to find the recyclables amongst the discarded materials. Under the environmental stewardship program, information about waste separation at the source is collected and the program teaches households about recycling and brings them in contact with the waste pickers that work in their neighborhood, in order to start waste separation at the source. The women involved in this program developed a pedagogical approach, where they conducted a survey and, at the same time, hold informative conversations with household members, they speak at schools and other community institutions, and they promote the idea about source separation during recreational activities in parks and at public recycling centers, where the community can deliver their recyclable materials.

**Figure 1.**
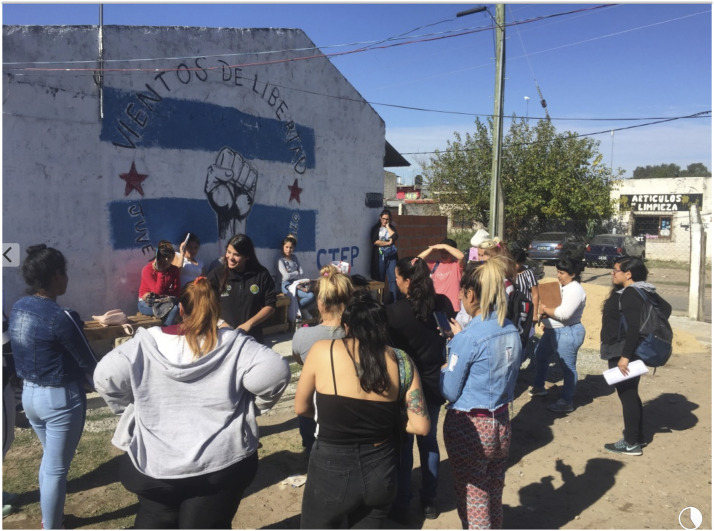
Workshop with waste pickers in Buenos Aires.

In September 2016, FACCyR who is committed to improving the livelihood conditions for all waste pickers in the country understood the importance of the role of the cooperative members as being the first link of the chain in a model of waste recovery, and they decided to replicate this experience with other cooperatives. In 2016, the researcher began working with FACCyR in two cooperatives, to whom the researcher established a trustful relationship, which reflects inconsistency in the support provided to the groups, the development of projects, and the stimulation of joint discussions and workshops to address the problems the cooperatives are facing (Figure 1). Based on their needs, they decided to start an environmental education course. During the courses, we noticed the need for a guide with systematized information on the course content, to be consulted in case they would have doubts. The idea was proposed to FACCyR, who was supportive, and together with the coordinator of the environmental promoters, the researcher started to develop a guide and course material.

Then, the researcher collaborated with many other courses to adapt these to the needs of the waste pickers in other cooperatives, also associated to FACCyR. The researcher took notes on key learnings, concepts, and methods from the course and passed them to the promoters as text. These notes allowed the systematization and the reformulation of the workshops and finally led to the development of the course material. The content of the courses is focused on four key themes:

• Introduction to the program of environmental stewards
• The challenge to separate waste at the source
• General concepts related to waste management systems
• Laws and regulations in waste management

The content was then discussed and collectively adjusted during follow-up meetings. Based on these courses, a guide was created, together with the development of a program for these courses. By doing so, not only the materials were developed but FACCyR also created a policy to set up these courses throughout the country with all cooperatives that are members of FACCyR. This social learning experience then also contributed to the creation of the training program itself.

Being a policy “made by women for women,” oriented towards waste pickers with a specific gender perspective and recognizing the work of women as a central part of the relationship between households and waste pickers, makes this program one of the foundational proposals for an Integrated Solid Waste Management Model with social inclusion, as part of the main demand of FACCyR’s platform, nationwide. Women were generally the ones who, during the door-to-door collection in the streets, established the links with the merchants and households. Being a woman made the communication easier, and strangers felt less intimidated than being contacted by men. The policy seeks to recognize the specific role of women and, at the same time, re-signifies their place in the work of collection and classification of recyclables. This Integrated Waste Management Model, where the promotion of source separation is a key issue, is being proposed in different places all around the country and, at the moment, it has been adopted by all members of the federation, as a central aspect of the work of waste pickers, not only to support their livelihoods through waste diversion but also as a way of rendering visible their work in the streets, in schools, sport clubs, and government offices.

### Waste Pickers Informing the Public in São Paulo, Brazil

The São Paulo case study, focuses on environmental education activities at the University *Fundação Centro Universitário Santo André*(FSA), where waste pickers have been invited to present in lectures and to participate in the dialogue with students and staff during the ‘Environment Week’ (Semanas Integradas do Meio Ambiente). The interventions of these waste pickers have challenged the everyday academic space and practices, traditionally perceived as symbol for authorized and legitimate knowledge development and expertise. This case study describes experiences invovlving two of the authors engaging with environmental education carried out by waste pickers in collaboration with academics from the University of Saaaaaa.

Since 1993, environmental education processes had been estabished at the institutional level, through specialization courses and other formal initiatives in this area. Between 2005 and 2011, teachers and students participated in a research and extension project (coordinated by the authors) aimed at strengthening waste pickers and public policies for waste management with social inclusion, a partnership between the University of Victoria – CA, the Faculty of Education at the University of São Paulo (USP) and is FEUSP, the Foundation, supporting USP, with a grant from the Canadian International Development Agency (CIDA). This partnership project called *Participatory Sustainable Waste Management* (PSWM) was developed using a PAR approach. Throughout the Project, exchanges of experiences and actions were developed in collaboration with waste pickers, contributing to their personal development, self-worth, internalization of cooperative values, and solidarity economy, and also with the appropriation of sociopolitical and environmental contexts (Baeder & Pontuschka, 2017). In 2006, PSWM supported the structuring of the first Environment Week, at the Fundação Santo André University Center, which then became an annual event, with an interdisciplinary character focused on the interaction with the community and society in general.

Furthermore, since 2011, waste pickers have been involved in giving talks and engaging in educational activities on recycling and related socio-environmental issues, specifically touching on waste challenges in the metropolitan region of São Paulo. During the lectures, the waste pickers’ stories highlight their origins and their everyday work experiences, pointing towards their struggles and difficulties to survive as well as their advances in organizing and creating new spaces as cooperatives, networks, and as a social movement.

The knowledge shared by these waste pickers include insights into technical aspects of the collection and separation of recyclable materials and their transportation, the organization and governance of work in cooperatives, the socio-environmental impacts caused by waste, as well as the necessity to prioritize waste avoidance and reduction. The waste pickers bring technical information with a critical positioning regarding the interests of their professional category and regarding the environmental impacts from waste which are a collective concern. Even given all the differences between the various speakers, over the years, their discourses emphasize the need for change in consumption behavior and the struggles to design public policies that are inclusive and tackle the social and environmental issues related to waste.

**Figure 2.**
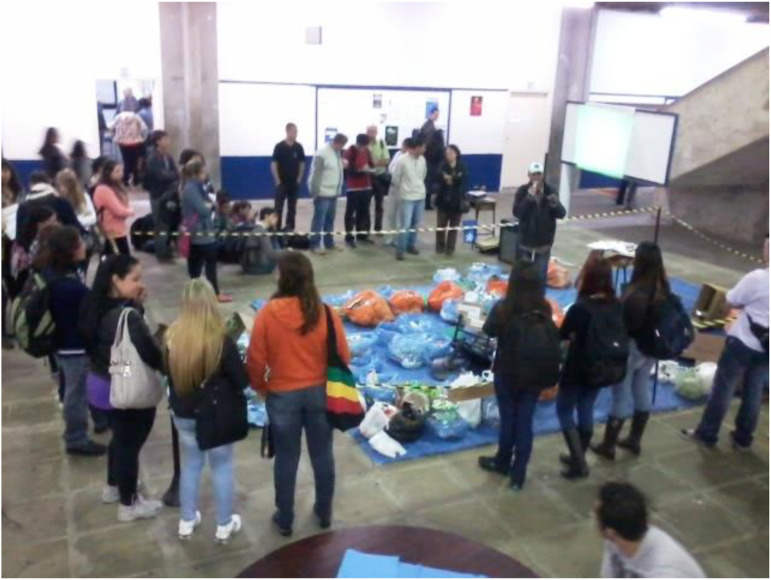
Intervention and waste exhibit at the university FSA, during the Environment Week in 2013.

In 2013, the Environment Week was carried out slightly different. The event was aimed at highlighting the need to implement selective waste collection and this intervention should help raise awareness among students, teachers, and employees on waste issues and the role of waste pickers. Piles of waste accumulated during the previous day was displayed in the universitys courtyard. The dialogue between waste pickers and the public developed as soon as the students left the classrooms and, along with their teachers and staff, visited the exhibit showing the garbage pile Figure 2. During this intervention, waste pickers posed provocative questions about waste generated within the university and how those left overs had their owners. They linked this scenario to waste disposal issues in Brazil, to the need for a greater commitment from all, in avoiding and reducing waste generation, in promoting adequate separation of waste materials, making waste infrastructures and services visible, and, therefore, making citizens confronting their waste, but also in participating in the struggles for social change towards more inclusive public policies. These issues were identified during the lectures, through written reports by students, and during the classes and activities that followed at the university.

These lectures and critical dialogues around the topic waste at the university have developed follow-up interventions. The waste pickers involved were able to sensitize those who were present, by their special way of approaching the topic and by their communication filled with lived knowledge, emotions, and meanings. The work and struggles of the waste pickers, hitherto ignored by the academic community, were thus revealed. In this interaction, the waste pickers themselves were also challenged by entering new spaces and speaking in these spaces from which they were previously excluded.

The agility performed by the waste pickers during these lectures indicates a possible contribution of the PSWM to their empowerment (as was also concluded by the Project’s independent final evaluation survey), which was later expanded throughout the networks of interactions between groups in the region as well as their participation in the National Waste Pickers Movement (MNCR), which they helped to strengthen. In parallel, the exchange of experience has supported the empowerment of waste pickers but has also helped make these organizations stronger. At the beginning of the PSWM project, a long process was required until the waste pickers felt at ease in the physical space of the university, which they never dreamed of having access to. Then during the events, they appeared as protagonists in the teaching of various types of knowledge. Our long term contact with waste pickers, dating to the beginning of 2005, allow us to perceive such changes.

Since then, the waste pickers have also been lecturing in different elementary schools, bringing to these spaces a reflection and awareness through the encounters with their real world, giving new meanings to the knowledge usually stripped of the historical complexity that engenders socioenvironmental problems.

The approach by the waste pickers to socioenvironmental themes at the university has instigated yet another important dimension, impregnated with meanings and social representations (Moscovici, 2003): regarding to what they think and feel about the world, as social subjects, and about their relationships with nature. Their values and reflections about the government, their superiors, co-workers, and about academic knowledges became evident. They contributed significantly to the debate with their knowledge about selective waste collection. These "technical issues" regain "life," that is, they become re-situated in a place where the supposed “scientific neutrality” had removed them from. They re-emerge, filled with values, shaped by the complexity of the social relations in their everyday life, giving them other, different meanings compared to those imposed by “neutral” observation.

Waste pickers' voices are usually not recognized within the Social Sciences, but through their narratives, the social, economic, cultural, political, and human dimensions of their exclusion becomes evident. In interactions, they demonstrate that beyond the work they do, they are also people, social subjects. This was a more frequent and relevant aspect that emerged out of the reports from the students delivered after the lectures. Since the first intervention, the awareness among students and faculty had increased and particularly students showed a higher level of interest and sensitivity to selective waste collection issues, attested by the surveys conducted after these Environment Weeks. In addition, the community’s response to institutional debates on the implementation of selective waste collection was greater which, in 2017, resulted in the implementation of the Selective Waste Collection Program at FSA. This accomplishment was not only the result of the lectures given by the waste pickers, but also of the reports and research confirming the importance of their contributions.

The following table (Table 1) summarizes key findings linked to our theoretical and analytical framework, which will be discussed in the next section.

**Table 1.** Waste Pickers Engaging in Educational Activities in Buenos Aires and São Paulo.

	Argentina	Brazil
Transformative space	Within the cooperative (*Anuillan*)	The University (*FSA*): classrooms and central hallway
Target population	Waste pickers and wider community	Students, faculty, staff, surrounding community
Immediate product of intervention or activity	Curriculum development	Environmental education with interventions to deconstruct waste and to question consumption
	Content development (training courses for recycling cooperatives in Buenos Aires)	
	Methodological guidebook (training manual)	
Insurgency aspect	Gender and empowerment	Entering spaces previously inaccessible, De-stigmatization, Valuing the work of waste pickers, Radical exposure to waste impacts
	Waste as a resource	
	De-stigmatization of waste pickers	
	Valuing the service of waste pickers	
	Local community focus	
Environmental stewardship aspect	Waste avoidance and waste reduction	Education on the work of waste pickers
	Efficient and effective resource recovery	Knowledge co-creation
Learning aspect	Recognition of waste pickers as environmental stewards	Responsible consumption, valuing reduction and reuse, correct waste disposal for recycling, waste as a resource, waste pickers as service providers
	Separation at the source	
	Waste management	

## Discussion

The collective impact of waste actors such as the waste pickers in Argentina and Brazil on improving the environment goes beyond the environmental and sanitation measures mandated by the government (McCay et al., 2014), and therefore, these actors engage with more sustainable waste regimes than the authorities themselves. Environmental promoters of the Argentinean Federation of Cartoneros, Carreros y Recicladores (FACCyR), aim to teach the neighbors in the city of Buenos Aires how to sort waste correctly. In this case, it is important to notice that one of the first struggles of the waste picker cooperatives in Buenos Aires was to dismantle the predominant waste regime which consisted of landfilling all waste generated in the city. The waste pickers were the first to advance and advocate for a radical change, showing the environmental and social importance of recycling. This knowledge was constructed during the first years of their intervention, when the problem was first put on to the public agenda. As a consequence, the perception of waste pickers as environmental promoters began to increase, focusing on women workers in particular. The connection between their local waste management practices and global discourses of environmental protection and recycling, led them to engage in a broader demand than just their labor needs. The framing of waste pickers as environmental stewards links the knowledge and the praxis of the waste pickers as resource reclaimers working in the city. By claiming the moral right to discards, the waste pickers’ movement recalls ideas of environmental stewardship (Lane & Watson, 2012) as well as Harvey’s “right to the city” (Harvey, 2012). It also recalls the right to discards as “commons” (Dias, 2016; Zapata Campos & Zapata, 2015), the commons being both the planetary natural environment and the discards as assets that are available for the urban poor. In the case of waste, these resources have for decades and worldwide been reclaimed as a livelihood strategy, by the waste pickers.

Our research also shows efforts to diffuse and institutionalize these knowledges towards mainstream institutions. In Argentina, a collaboration with FACCyR works towards the development of a training manual and the systematization and programming of a course that can be replicated in different cooperatives throughout the country, thus expanding the impact of waste pickers’ environmental stewardship education. This process is also essential for the construction and consolidation of new paradigms. In the two cities, the institutionalization of these knowledges encountered a “porous” and receptive public administration (be it the city or the university administration) (Zapata Campos & Zapata, 2017) that facilitated infiltration strategies through which moderately these novel practices could be introduced. The two initiatives deal with the construction of new values and rationalities through practical activities and new interactions that penetrate and alter the dynamics of the personal lives and cities in general. Material, social, and environmental rationales are entangled and difficult to separate (Gutberlet et al., 2017) and together fuel its penetration to further these groups of insurgent citizens (Zapata Campos & Zapata, 2017).

In the case of the environmental stewards in Buenos Aires, it was a bottom-up approach, with the waste pickers themselves being the ones that implemented the training with waste pickers, and not people from the outside, contracted by the government. In this way, they were able to fight for recognition, both of their work and of their specific knowledge regarding recycling. In the case of Brazil, while initiated with support from the university, waste pickers continued on their own, promoting environmental education at schools and among clients (small business and housing communities), giving presentations and engaging in meetings, often involving struggles and disputes in order to access new spaces and to de-stigmatize their activity.

Ultimately these interventions, such as the waste related activities with waste pickers during the Environment Week at the university FSA in São Paulo have created the potential for generating more sustainable ways of producing, consuming, and living. Same results are achieved by the environmental stewardship of informed waste pickers in Buenos Aires who have been trained and educated to disseminate best material separation practices at the source. In the case of Buenos Aires, in buildings where only 20% of the housing units separated their waste, the number rose to 80% after the intervention of the environmental stewards. The interactions between waste pickers and university students, schools, ordinary citizens, politicians, and public officers, as well as with members from other organized waste picker cooperatives and networks are geared towards endurable changes in relation to solid waste and related socioenvironmental aspects, facilitating the transition between local scale and more structural societal changes (Smith et al., 2017). In both cases, waste pickers educate citizens for adequate separation of waste materials, making waste infrastructures and services visible (Star, 1999) and making everybody confronting their waste (Hawkins, 2017). With the diffusion of their practices, the waste picker movements modify the assumptions and practices of consumption, discard, and recovery of natural resources. In other words, they question the existing relations people have with their own waste, creating opportunities to revaluing materials and change consumer behavior (Hawkins, 2017), therefore generating the insurrection of subjugated knowledges, which means “knowledges that have been disqualified as inadequate to their task” (Foucault, 1980, p. 81–82).

In the interactions between schools and waste pickers, described in the Brazilian case study, new habits and attitudes are created, enabling the *capillarization*, the rooting of these learnings, reflecting in general perceptions and public policies related to environmental education (Brazil, 2012). This capillarization was possible by adopting a “waste picker’s gaze.” As Zapata and Zapata Campos (2018) have shown in the case of waste tours in Sweden, tour guides can direct the visitor’s gaze towards certain issues, values, and practices, while other can vanish from the framing of the scenario. Here, we argue that a “waste picker’s gaze” is fundamental to engage citizens and residents in more sustainable recycling practices. Waste pickers’ knowledge based on their participation in this community of practice is crucial. The experiences of the waste pickers bring meaning and content to the practice of recycling and waste management, just as Paulo Freire highlights the fact that educating is not just the transferal of knowledge, but rather a praxis where theory and “**concrete, practical examples of theory come together***.* …*I* [as a teacher]**must already be involved in it** *…*and where the praxis and theory **also needs to be constantly witnessed, lived***”* (Freire, 1997, p. 21). That is the strength of waste pickers in their role as insurgent environmental stewards. The waste pickers are able to promote social learning, bringing the participants closer to the quest for social and environmental sustainability, stimulating participation in the management and solution of problems, both in schools and universities and potentially also in the wider urban community (Baeder, 2009; Loureiro, 2004). Through interventions and communications, waste pickers in both cases are acting as environmental stewards, teaching about the advantages of recycling, showing the value chains of the materials, and also giving an account of the social inclusion that is achieved through the recognition of the work of waste pickers.

Waste pickers are vulnerable to shifts in politics, particularly after elections. Political will may change and previously supported programs for waste pickers can quickly become discontinued as a consequence. Previously valued environmental education activities performed by waste pickers become dismantled. Newly elected governments often decide to oppose preceding programs and terminate relations and policies that had funded waste pickers’ until then (Sorroche, 2018).

Regarding the Brazilian case study, it is important to point out that the relations with the municipal governments vary greatly from one city to another and, similarly, the interactions between the political leaders and cooperative members. It would be very positive to strengthen both internal relations and the relations of different governments with cooperatives, in order to achieve better conditions, especially for those that are still fragile. However, it is possible to emphasize the aspect that there was a noticible advance in the concrete conditions and in the expansion of knowledge and action towards changing the current conditions.

Waste pickers challenge notions of modernity, for example, how the latest, largest, and more expensive technology might not necessarily be the most appropriate one. These notions of modernity are deeply entrenched in waste management governance, and achievements gained after years of struggles by these groups can be quickly eroded. On that note previous research has shown how local politicians and municipal officers observe these low-tech grassroots solutions as temporary until “modern” appropriate technologies are available or can reach, for example, informal settlements; after they are upgraded, their streets widened and cables lift up enough to allow a modern waste truck to enter them (Zapata Campos & Zapata, 2014). Following Foucault, the insurrection of subjugated knowledges (including those from other individuals, e.g., local residents) generates “alternatives to modernity,” as informal settlements challenge the ideal of modernity, the ideal of a modern and rational city that planners and politicians envision in their plans. Insurgency actions do not necessarily persist as insurgent citizens, and social movements weaken or give up.

Exclusion takes place in an objective and material way. Waste pickers often work under precarious conditions from childhood on to maintain themselves. They are usually unable to buy books or cloths and other material goods. Their exclusion also takes place in a subjective and symbolic form, influenced by values, religiosity, devalued empirical knowledge, among other intangible cultural aspects, as illustrated by Bourdieu et al (1999) through “cultural capital” as a form of domination. The presentations of the waste pickers in the described cases revealed an amalgamation between technical aspects of selective waste collection, the historical issues related to waste picking, as well as the material and immaterial needs for their survival. Yet, precisely the insurrection of this subjugated knowledge serves to invert the identity of the excluded.

Socio-environmental issues come to life when contextualized in everyday experiences. The ideological dimension and the political clashes arising from socio-environmental interactions between waste pickers and other social actors become evident and shape the content and process of environmental education. In the presence of waste pickers, reality is loaded with the possibility of acting in order to change the socio-environmental condition and overcome the immobility and apathy in the face of the amplitude of the problems (Sorrentino et al., 2001). Students, teachers, and employees have their own agency and the potential to act and seek socio-environmental sustainability, breaking the paradigm of society’s relationship to nature, based on profit-oriented exploitation of resources, mass consumerism, and the perpetuation of social differences. The described interventions of waste pickers as environmental stewards, as they happen in São Paulo, Buenos Aires, and elsewhere can break with the paradigm, as expressed by Tião Santos, president of the waste picker association of the metropolitan landfill in Jardim Gramacho (Associação dos Catadores do Aterro Metropolitano de Jardim Gramacho) in Rio de Janeiro: "People consume too much and is so exaggerated and unnecessary, and the worst is that people don’t think ... I mean everyone, everywhere in the world takes their garbage and puts it in the door of their homes ... and then the garbage disappears. ... People think that it evaporates, that it does not occupy a place in space ... that it will decompose in the environment. So, I think before people consume, they should think about recycling too ....we need to consume in a sustainable way, in a conscious way" (Gutberlet et al., 2009).

In both cases, actors from universities were involved in supporting the creation of small spaces of experimentation, producing knowledge at the margins. In some cases, these became innovative spaces, creating, for example, a transformative curriculum with courses appropriate to the needs of the waste pickers as well as a methodological guidebook providing orientation about the training and learning program developed by the waste pickers. In the Brazilian case, opening up the university space for insurgent interventions by the waste pickers had stimulated reflections over waste and resource recovery and how to create a more just society, valuing the work of waste pickers. Both examples highlight the fact that here we are producing knowledge from the margins at least in two ways: the margins of cities, as many waste pickers serve informal settlements and low-income neighborhoods; and the margins of the system, as literally waste management (still) holds a place at the end of the pipeline in our society. In addition to the new knowledge produced, the studies reveal a change in attitude of these waste pickers in the direction of personal transformations, as evidenced by the very fact that they took responsibility for the performance of lectures and actions in which they became protagonists.

## Conclusions

In this paper, we argue for the expansion of the concept of local environmental stewardship to include the insurgent perspective of grassroots initiatives who get involved in diverse innovative, educational contributions. Insurgent citizenship involves those that manage to innovate amid the necessity of coping with urban inequalities (Friendly and Stiphany, 2019). The integration of stewardship considerations into local waste management programs supports planned or anticipated interventions, involving the praxis and understandings of waste pickers.

The examples from Argentina and Brazil demonstrate how individuals who have historically been excluded and stigmatized, are speaking up, reinventing themselves as insurgent citizens and environmental stewards, making themselves heard in spaces they were previously not invited to, as, for example, the university. The cases serve to illustrate how knowledge production can be insurgent being co-created from the periphery of the systems by questioning the current culture of consumption and discard, giving the power to recover resources by ordinary people.

Insurgent citizenship challenges established knowledges, questions technology assumptions linked to modernity, and critiques the concept of “the expert,” sometimes creating clashes with more powerful actors (politicians, professors, university administrators, etc.) in these newly occupied spaces. The learning that stems from these grassroots processes is insurgent. Insurgent learning is experiential and validated by everyday experiences of subaltern populations.

In the case study from São Paulo, Brazil, the lectures allowed for the creation of a bond between students, teachers, and staff with the socio-environmental situation experienced by waste pickers, a social group largely excluded in all dimensions of life. In part, the lectures filled the frequent "void" between academia and reality, as observed in traditional research and even extension work, which often acts as "deposition of knowledge in communities" and understands social subjects as simple “study goals” (Green, 2008). Ideally, this open “dialogue” with the community should be expanded, transversal, and continued, encompassing various areas of academic knowledge and, on the other hand, interacting with other social categories and other socio-environmental problems. This would enlighten research and bring new ideas to overcome challenges in society.

The knowledge of waste pickers allows for policy recommendations supporting other forms of interventions in waste management that have the potential to be most beneficial to the urban context. Their suggestions to the government include the need to commit to capacity building of waste pickers to implement door-to-door separate material collection and household education for recycling on an extensive scale. Anchored in public policies that guarantee continuous environmental education and awareness building, the quality of source separation and the recycling rates can be improved. Waste pickers also request to participate in reverse logistics schemes. EPR policies need to recognize waste picker organization as important actors in the circular economy. Finally, waste pickers require resources, technology, and access to knowledge to further improve their capacity to work in formal waste management and environmental education using their real life experiences with waste to leverage for greater awareness of the general public. From a socioecological perspective, waste pickers are important actors with the potential to address some of the most pressing waste related issues in the city, reducing waste leakage into the environment, increasing the circular economy, and promoting social inclusion.

We agree with Bennett et al. (2018) on the assumption that “NGOs, local organizations, co-management bodies, or informal networks—can provide reinforcement for local collective actions, generate resources or facilitate learning for stewardship” (2017, p. 601). However, the still neglected conditions under which most waste pickers have to work are a huge impediment in their strive as environmental stewards. While the waste education interventions described here have the potential to address the quest for more social and environmental justice outcomes, insurgency is a cutting edge and often extremely vulnerable undertaking, not always successful in promoting the aspired outcomes of change. Waste pickers learn through their everyday livelihood struggles, fighting poverty, oppression, discrimination, stigmatization, exclusion, precarious and unhealthy living conditions, and other injustices. They learn about waste from a different perspective than the rest of the society, often living in neighborhoods with no regular waste collection, making a living from resources embedded in waste and living the drama of littered waste causing water logging affecting their communities with floods and disease. Insurgent learning is powerful and challenges existing paradigms. The specific learning that happens through daily routines and what is considered ordinary as it refers to subaltern populations feeds into social movement learning, of which many of the waste pickers in Brazil and Argentina are part of. However, up to date waste governance and specifically the policy realm does not yet recognize the contribution of the knowledge produced by waste pickers, and there are no formal structures in place to expand, disseminate, and implement the grassroots learning around the topic waste on an expanded scale.

In this article, we have discussed the emergent spaces of invention and agency where waste pickers become active. New kinds of active citizens and citizenships emerge in global peripheral urbanization, as suggested by Holston (2009). We demonstrate, by combining the concepts of insurgent citizenship with environmental stewardship, how grassroot initiatives have contributed towards wasting less and building a more just society.

Finally, the interaction between knowledge produced in academia and local knowledge is always a reason for reflection, especially when the aim is to empower and expand the autonomy of exploited community social actors (ANTEAG, 2005). Access to scientific information and technologies is a “right for everyone,” but one that is extinguished amidst inequalities. The challenge is to promote access, to seek equality, and to appreciate other knowledges. The dialogue with waste pickers and the amalgamation of knowledges are necessary to overcome current problems. Although the required transformations seem utopian and unattainable, reflecting on them makes us walk and strive to accomplish change (Galeano, 1994). Insurgent movements are fundamental to breaking through and seeking new conditions.

## References

[bibr82-10704965211055328] Amauchi J. Gauthier M. Ghezeljeh Z. Giatti L. Keats K. Sholanke D. Zachari D. Gutberlet J. (2021). The power of community-based participatory research: ethical and effective ways of researching. Community Developmen. 10.1080/15575330.2021.1936102.

[bibr1-10704965211055328] ANTEAG (2005). Autogestão e Economia Solidária: uma nova metodologia (Vol. 2). Brasília: Ministério do Trabalho e Emprego: Secretaria de Políticas Públicas de Emprego.

[bibr2-10704965211055328] Asmawati D. AbdKadir N. B. Yusooff F. (2012). Environmental awareness and education: A key approach to solid waste management (SWM) – a case study of a university in Malaysia. In Waste Management - An Integrated Vision, Luis Fernando Marmolejo Rebellon. DOI: 10.5772/48169.

[bibr3-10704965211055328] Baeder A. M. (2009) Educação Ambiental e Mobilização Social: Formação de Catadores na Grande São Paulo. [Thesis Doctor in Education, Faculdade de Educação da Universidade de São Paulo], São Paulo.

[bibr4-10704965211055328] Baeder A. M. Pontuschka N. N. (2017) Educação Ambiental e Mobilização com catadores de materiais Recicláveis: Solução de problemas, resgate de autoestima e construção de autonomía. In Libro del 2° Encuentro Internacional La Formación Universitaria y la Dimensión Social del Profesional: a 46 años del Taller Total en la UNC /CaioBoucinhas ... [et al.]; compiled by Simone Rocha de Abreu; Nora Zoila Lamfri; Sylvia Adriana Dobry & Maria Sabina Uribarren (1st ed.), Córdoba: Universidad Nacional de Córdoba.

[bibr5-10704965211055328] Bennett N. J. Whitty T. S. Finkbeiner E. Pittman J. Bassett H. Gelcich S. Allison E. H. (2018) Environmental stewardship: a conceptual review and analytical framework. Environmental Management, 61(4), 597-614. 10.1097/10.1007/s00267-017-0993-2. 29387947 PMC5849669

[bibr6-10704965211055328] Bourdieu P. (1999) Escritos de educação. Catani, M. A. & Catani, A. (Org.), Petrópolis, RJ (2nd ed.). Vozes (pp. 71-79).

[bibr7-10704965211055328] Boyes E. Stanisstreet M. (2012). Environmental education for behaviour change: which actions should be targeted? International Journal of ScienceEducation, 34(10), 1591-1614. 10.1097/10.1080/09500693.2011.584079

[bibr8-10704965211055328] Brandão C. R. (1987). Pesquisar-participar. In Brandão C. R. (Ed.), Repensando a pesquisa participante (3rd ed., pp. 7–14). São Paulo, Brazil: Brasiliense.

[bibr9-10704965211055328] Brazil (2012). Passo a passo para a Conferência de Meio Ambiente na Escola + Educomunicação: escolas sustentáveis / Grácia Lopes, Teresa Melo e Neusa Barbosa. – Brasília: Ministério da Educação, Secadi: Ministério do Meio Ambiente, Saic. 56p.

[bibr10-10704965211055328] Brown H. S. Vergragt P. J. (2008). Bounded socio-technical experiments as agents of systemic change: The case of a zero-energy residential building. Technological Forecasting & Social Change*,* 75(1), 107–130. 10.1016/j.techfore.2006.05.014

[bibr11-10704965211055328] Corvellec H. (2016). Waste management: the other of production, distribution, and consumption. In Czarniawska B. (Ed.) A research agenda for management & organization studies (pp. 107-114). Cheltenham (UK): Edward Elgar.

[bibr12-10704965211055328] Corvellec H. Hultman J. (2012). From “less landfilling” to “wasting less”: societal narratives, socio-materiality, and organizations. Journal of Organizational Change Management, 25(2), 297–314. 10.1108/09534811211213964

[bibr13-10704965211055328] Corvellec H. Zapata Campos M. J. Zapata P. (2013). Infrastructures, lock-in, and sustainable urban development: the case of waste incineration in the Göteborg Metropolitan Area, Journal of Cleaner Production, 50(1), 32–39. 10.1016/J.JCLEPRO.2012.12.009

[bibr14-10704965211055328] Demaria F. Schindler S. (2016). Contesting urban metabolism: struggles over waste‐to‐energy in Delhi, India. Antipode, 48(2), 293-313. 10.1111/anti.1219

[bibr15-10704965211055328] Dias S. (2011). Women in informal employment: globalizing and organizing. WIEGO Policy Brief. Urban Policies, 5, 8.

[bibr16-10704965211055328] Dias S. M. (2016). Waste pickers and cities. Environment & Urbanization, 28(2), 375–390. 10.1177/0956247816657302

[bibr17-10704965211055328] Eyerman R. Jamison A. (1991) Social movements: A cognitive approach. Oxford: Polity Press.

[bibr18-10704965211055328] Foley G. (1999). Learning in social action. LondonLeicester: Zed Books (with NIACE).

[bibr19-10704965211055328] Fahmi W. Sutton K. (2010). Cairo’s contested garbage: sustainable solid waste management and the Zabbaleen’s right to the city. Sustainability, 2, 1765-1783. 10.3390/su2061765

[bibr20-10704965211055328] Foucault M. (1980) Power/knowledge: Selected interviews and other writings 1972*-*1977 New York: Pantheon.

[bibr21-10704965211055328] Freire P . (1997) Pedagogia da autonomia: Saberes necessários à prática educativa. São Paulo: Editora Paz e Terra, (Coleção Leitura), 165.

[bibr22-10704965211055328] Friendly A. Stiphany K. (2019). Paradigm or paradox? The ‘cumbersome impasse’ of the participatory turn in Brazilian urban Planning. Urban Studies, 56(2): 271-287. 10.1177/0042098018768748

[bibr23-10704965211055328] Frisby W. Maguire P. Reid C. (2009). The “f” word has everything to do with it: How feminist theories inform action research. Action Research, 7(1), 13-29. 10.1177/1476750308099595

[bibr24-10704965211055328] Gaia (2021) Gaia homepage. https://www.no-burn.org/

[bibr25-10704965211055328] Galeano E . (1994) Las palavras andantes. Madrid: Siglo XXI.

[bibr26-10704965211055328] Gaudry A. (2015). Researching the resurgence: insurgent research and community-engaged methodologies in 21st-century academic inquiry. In Brown S. Strega L. (Eds.), Research as Resistance. Revisiting Critical, Indigenous, and Anti-opressive approaches (pp. 243-265). Canadian Scholars’ Press.

[bibr27-10704965211055328] Gille Z. (2013). From risk to waste: global food waste regimes. The Sociological Review 60(S2), 27-46. 10.1111/1467-954X.12036

[bibr28-10704965211055328] Green R. (2008). Bringing about social change: the role of community research. In Cox P. Geisen T. Green R. (Eds.), Qualitative research and social change. european contexts (pp. 75-93). New York: Palgrave and Macmillan.

[bibr29-10704965211055328] Gulrajani N. Swiss L. (2019). Donorship norms in a state of flux In Olivié I. Pérez A. (Eds.), Aid power and politics (pp. 199-222)*,* London: Routledge.

[bibr30-10704965211055328] Gutberlet J. (2010). Waste, poverty and recycling. Waste Management, 30(2), 171-173. 10.1016/j.wasman.2009.11.006 19962085

[bibr31-10704965211055328] Gutberlet J. (2016). Ways out of the waste dilemma: transforming communities in the global south. In Mauch C. (Ed.), A future without waste? zero waste in theory and practice (Vol. 3, pp. 55-68) RCC perspectives: Transformations in Environment and society. http://www.environmentandsociety.org/perspectives/2016/3/future-without-waste-zero-waste-theory-and-practice

[bibr32-10704965211055328] Gutberlet J. Bramryd T. Johansson M. (2020). Expansion of the waste-based commodity frontier: insights from Sweden and Brazil. Sustainability, 12(7) 2628. 10.3390/su12072628

[bibr33-10704965211055328] Gutberlet J. Kain J. -H. Nyakinda B. Oshieng D. H. Odhiambo N. Oloko M. Omolo J. Omondi E. Otieno S. Zapata P. Zapata Campos M. J. (2016). Socio-environmental entrepreneurship and the provision of critical services in informal settlements. Environment and Urbanization, 28(1), 205-222. 10.1177/0956247815623772

[bibr34-10704965211055328] Gutberlet J. Kain J.-H. Nyakinya B. Oloko M. Zapata P. Zapata Campos M. J. (2017). Bridging weak links of solid waste management in informal settlements. The Journal of Environment & Development, 26(1), 106-131. 10.1177/1070496516672263

[bibr35-10704965211055328] Gutberlet J. Tremblay C. Searl R. (2009). Beyond Gramacho (Video Documentary - English). www.JuttaGutberlet.com (Video)*.*

[bibr36-10704965211055328] Hall B. Clover D. Crowther J. Scandrett E. (2005). Learning and education for a better world: The role of social movements. Rotterdam: Sense Publishers.

[bibr37-10704965211055328] Harvey D. (2012). Rebel cities: From the right to the city to the urban revolution. London: Verso.

[bibr38-10704965211055328] Hawkins G. (2017). Ethical blindness: plastics, disposability and the art of not caring In Kinnunen V. Valtonen A. (Eds), Living ethics in a more-than-human world (pp. 15–28). Finland: University of Lapland.

[bibr39-10704965211055328] Hird M. (2013). Waste, landfills, and an environmental ethic of vulnerability. Ethics & the Environment, 18(1), 105-124. 10.2979/ethicsenviro.18.1.105

[bibr40-10704965211055328] Hird M. J. Lougheed S. Rowe K. Kuyvenhoven C. (2014). Making waste management public (or Falling Back to Sleep), Social Studies of Science, 44(3), 441-465. 10.1177/0306312713518835 25051590 PMC4509873

[bibr41-10704965211055328] Holston J. (2008). Insurgent citizenship: Disjunctions of democracy and modernity in Brazil*.* Princeton, NJ: Princeton University Press.

[bibr42-10704965211055328] Holston J. (2009). Insurgent citizenship in an era of global urban peripheries. City & Society, 21(2), 245-267. 10.1111/j.1548-744X.2009.01024.x

[bibr43-10704965211055328] Jupiter S. D. Cohen P. J. Weeks R. Tawake A. Govan H. (2014). Locally-managed marine areas: multiple objectives and diverse strategies. Pacific Conservation Biology, 20(2), 165–179. 10.1071/PC140165

[bibr83-10704965211055328] Kaza S. Yao L. Bhada-Tata P. Van Woerden F. (2018). What a waste 2.0: A global snapshot of solid waste management to 2050. Urban Development Series. World Bank Publications.

[bibr44-10704965211055328] Kidd S. A. Kral M. J. (2005). Practicing participatory action research. Journal of Counselling Psychology, 52(2), 187-195. 10.1037/0022-0167.52.2.187

[bibr45-10704965211055328] Kindon S. (2016). Empowering approaches: participatory action research. In Hay I. (Ed.), Qualitative Research Methods in Human Geography (pp. 350-370). Oxford University Press.

[bibr46-10704965211055328] Lane R. Watson M. (2012). Stewardship of things: the radical potential of product stewardship for re-framing responsibilities and relationships to products and materials. Geoforum, 43(6), 1254-1265. 10.1016/j.geoforum.2012.03.012

[bibr48-10704965211055328] Lepawsky J. (2018). Reassembling rubbish worlding electronic waste. Cambridge, MA: The MIT Press.

[bibr49-10704965211055328] Lockwood M. Davidson J. Curtis A. Stratford E. Griffith R. (2010) Governance principles for natural resource management. Society and Natural Resources 23, 986–1001. 10.1080/08941920802178214

[bibr50-10704965211055328] Loureiro C. F. B. (2004) Educação ambiental transformadora. In: Layargues P. P. Identidades da educação ambiental brasileira. Diretoria de Educação Ambiental. Brasília: Ministério do Meio Ambiente. 155 pp.

[bibr51-10704965211055328] MacBride S. (2012). Recycling reconsidered: the present failure and future promise of environmental action in the United States. Cambridge, MA: The MIT Press.

[bibr52-10704965211055328] McCay B. J. Micheli F. Ponce-Díaz G. Murray G. Shester G. Ramirez-Sanchez S. Weisman W. (2014) Cooperatives, concessions, and co-management on the Pacific coast of Mexico. Marine Policy*,* 44, 49-59. 10.1016/j.marpol.2013.08.001

[bibr53-10704965211055328] McConney P. Medeiros R. Pena M. (Eds.) (2014) Enhancing Stewardship in Small-Scale Fisheries: Practices and perspectives. Too Big To Ignore (TBTI) and Centre for Resource Management and Environmental Studies, The University of the West Indies, Cave Hill Campus, Barbados. CERMES. TechnicalReport No. 73. 162p.

[bibr54-10704965211055328] Medeiros R. P. Serafini T. Z. McCooney P. (2014) Fortalecendo o ecosystemstewardship na pesca artesanal: perspectivas para a América Latina e Caribe. Desenvolvimento e MeioAmbiente, 32, 181-190. 10.5380/dma.v32i0.38819

[bibr55-10704965211055328] Miraftab F. (2004). Invented and invited spaces of participation: neoliberal citizenship and feminists’ expanded notion of politics. Wagadu: 1: 1-7. http://colfax.cortland.edu/wagadu/Volume%201/Printable/miraftab.pdf

[bibr56-10704965211055328] Miraftab F. (2009). Insurgent planning: situating planning in the global south. Planning Theory 8(1), 32–50. 10.1177/1473095208099297

[bibr57-10704965211055328] Mitlin D. (2008). With and beyond the state: co-production as a route to political influence, power and transformation for grassroots organizations. Environment and Urbanization, 20(2), 339-360. 10.1177/0956247808096117

[bibr81-10704965211055328] Moscovici S. (2003). Representações sociais: investigações em psicologia social. Rio de Janeiro: Vozes.

[bibr58-10704965211055328] O’Brien M. (2008). A crisis of waste? Understanding the rubbish society, New York: Routledge.

[bibr59-10704965211055328] Pandya R. E. (2012). A framework for engaging diverse communities in citizen science in the US. Frontiers in Ecology and the Environment, 10(6), 314-317. 10.1890/120007

[bibr60-10704965211055328] Parizeau K. (2015). Re-representing the city: Waste and public space in Buenos Aires, Argentina in the late 2000s. Environment and planning A: Economy and space, 47(2), 284-299. 10.1068/a130094p

[bibr61-10704965211055328] Santoro Franco M. A. (2005). Pedagogia da pesquisa-ação (Pedagogy of action-research). Educação e Pesquisa, 31(3), 483–502. 10.1590/S1517-97022005000300011

[bibr62-10704965211055328] Simone A. (2010). A town on its knees? Economic experimentations with postcolonial urban politics in Africa and Southeast Asia. Theory, Culture, Society*,* 27(7-8) 130-154. 10.1177/0263276410383708

[bibr63-10704965211055328] Sjöstrand Y. (2014). Stadens sopor: Tillvaratagande, förbränning och tippning i Stockholm 1900-1975, Lund: Nordic Academic Press.

[bibr64-10704965211055328] Smith A. Fressoli M. Abrol D. Arond E. Ely A. (2017). Grassroots innovation movements. Pathways to sustainability. London: Earthscan.

[bibr65-10704965211055328] Smith A. Fressoli M. Thomas H. (2014). Grassroots innovation movements: challenges and contributions. Journal of Cleaner Production, 63, 114-124. 10.1016/j.jclepro.2012.12.025

[bibr66-10704965211055328] Sorrentino M. Sposati A. Sawaia B. Dalari D. et al. (2001). Ambientalismo e participação na contemporaneidade. São Paulo: EDUC/FAPESP.

[bibr68-10704965211055328] Sorroche S. (2016b). Ni “vagos” ni “ladrones”: trabajadores cartoneros. Las organizaciones cartoneras y la disputa por el reconocimiento de su actividad como un trabajo. *Épocas*. Revista de Ciencias Sociales y Crítica Cultural, 3.

[bibr67-10704965211055328] Sorroche S. (2016a). Gubernamentalidad global y vernaculización en la gestión de residuos. Análisis etnográfico desde la experiencia de cooperativas de cartoneros en el Gran Buenos Aires*.* Tesis de Doctorado. Facultad de Filosofía y Letras, Universidad de Buenos Aires.

[bibr69-10704965211055328] Sorroche S. (2018). Vinculaciones entre gobiernos municipales y cooperativas de cartoneros. Análisis de dos casos del Gran Buenos Aires. In Suárez F. Y. Schamber P. (Eds.), Recicloscopio V*.* Buenos Aires: UNQ/UNLa./Ediciones UNGS.

[bibr70-10704965211055328] Star S. L. (1999). The Ethnography of Infrastructure, American Behavioral Scientist*,* 43(3), 377. 10.1177/00027649921955326

[bibr71-10704965211055328] Strasser S. (1999). Waste and want: A social history of trash. New York: Metropolitan Books.

[bibr72-10704965211055328] Thiollent M. (2008). Metodologia da Pesquisa-Ação (Action-research methodology) (16th Ed.). São Paulo, Brazil: Cortez.

[bibr73-10704965211055328] Troschinetz A. Mihelcic J. R. (2009). Sustainable recycling of municipal solid waste in developing countries, Waste Management, 29(2), 915-923. 10.1016/j.wasman.2008.04.016 18657963

[bibr74-10704965211055328] Turner R. A. Fitzsimmons C. Forster J. Mahon R. Peterson A. Stead S. M. (2014) Measuring good governance for complex ecosystems: Perceptions of coral reef-dependent communities in the Caribbean. *Global Environmental Change* 29:105–117. 10.1016/j.gloenvcha.2014.08.004.

[bibr75-10704965211055328] Wenger E. (2010). Communities of practice and social learning systems: the career of a concept. In Blackmore C (Ed.), Social learning systems and communities of practice (pp. 179-198). London: Springer.

[bibr76-10704965211055328] Zapata Campos M. J. Zapata P. (2012). Changing la chureca. organizing city resilience through action nets, Journal of Change Management, 12(3), 323-337. 10.1080/14697017.2012.673073

[bibr77-10704965211055328] Zapata Campos M. J. Zapata P. (2014). The travel of global ideas of waste management. The case of Managua and its informal settlements. Habitat International, 41(1), 41-49. 10.1016/j.habitatint.2013.07.003

[bibr78-10704965211055328] Zapata Campos M. J. Zapata P. (2017). Infiltrating citizen-driven initiatives for sustainability. Environmental Politics, 26(6), 1055-1078. 10.1080/09644016.2017.1352592

[bibr79-10704965211055328] Zapata P. Zapata Campos M.J. (2015). Producing, appropriating and recreating the myth of the urban commons. In Borch C. Kornberger M. (Eds), Urban commons. Rethinking the city (pp. 92-108). New York: Routledge.

[bibr80-10704965211055328] Zapata P. Zapata Campos M. J. (2018). Waste tours: narratives, infrastructures and gazes in interplay. Etnografia Ricerca Qualitativa, 11(1), 97-118. 10.3240/89696.

